# Cardiac Innervation and Sudden Cardiac Death

**DOI:** 10.2174/157340309789317904

**Published:** 2009-11

**Authors:** Masaki Ieda, Keiichi Fukuda

**Affiliations:** Department of Regenerative Medicine and Advanced Cardiac Therapeutics, Keio University School of Medicine, 35 Shinanomachi, Shinjuku-ku, Tokyo 160-8582, Japan

**Keywords:** Cardiac nerve, nerve growth factor, Sema3a, arrhythmia, silent myocardial ischemia, sudden cardiac death.

## Abstract

The heart is extensively innervated and its performance is tightly controlled by the nervous system. Cardiac innervation density varies in diseased hearts leading to unbalanced neural activation and lethal arrhythmia. Diabetic sensory neuropathy causes silent myocardial ischemia, characterized by loss of pain perception during myocardial ischemia, which is a major cause of sudden cardiac death in diabetes mellitus (DM). Despite its clinical importance, the mechanisms underlying the control and regulation of cardiac innervation remain poorly understood.

We found that cardiac innervation is determined by the balance between neural chemoattractants and chemorepellents within the heart. Nerve growth factor (NGF), a potent chemoattractant, is induced by endothelin-1 upregulation during development and is highly expressed in cardiomyocytes. By comparison, Sema3a, a neural chemorepellent, is highly expressed in the subendocardium of early stage embryos, and is suppressed during development. The balance of expression between NGF and Seme3a leads to epicardial-to-endocardial transmural sympathetic innervation patterning. We also found that downregulation of cardiac NGF leads to diabetic neuropathy, and that NGF supplementation rescues silent myocardial ischemia in DM. Cardiac innervation patterning is disrupted in Sema3a-deficient and Sema3a-overexpressing mice, leading to sudden death or lethal arrhythmias. The present review focuses on the regulatory mechanisms underlying cardiac innervation and the critical role of these processes in cardiac performance.

## INTRODUCTION

Cardiac innervation density is altered in diseased hearts, as in cases of congestive heart failure and myocardial infarction [1-3]. Following myocardial injury, cardiac nerves undergo denervation, which may be followed by Schwann cell proliferation and reinnervation, leading to heterogeneous patterns of innervation [4, 5]. Abnormal sympathetic innervation may trigger lethal arrhythmia through ion channel modulation in cardiomyocytes [3, 6, 7]. In fact, there is circadian variation in the frequency of sudden cardiac death (SCD) that parallels sympathetic activity. β-blocker therapy prevents SCD secondary to ventricular tachyarrhythmia in ischemic heart disease or congestive heart failure [8, 9]. Immunohistochemical analysis of cardiac nerves in explanted hearts of transplant recipients reveals a positive correlation between nerve density and the clinical history of ventricular tachyarrhythmia [3]. Cardiac sensory denervation can also cause silent myocardial ischemia, characterized by loss of pain perception during myocardial ischemia and frequently leading to SCD in patients with diabetes mellitus (DM) [10]. Despite the severity of these complications, the molecular mechanism that determines innervation density in diseased hearts is poorly understood. Moreover, little is known about the anatomical distribution of cardiac nerves and the molecular mechanism regulating innervation during development [11]. The present review focuses on the regulatory mechanisms underlying cardiac innervation and the critical role of these processes in cardiac performance.

## ANATOMY OF CARDIAC NERVOUS SYSTEM

The cardiac sympathetic nerves extend from the sympathetic neurons of stellate ganglia, which are located bilateral to the vertebra. Sympathetic nerve fibers project from the base of the heart into the myocardium, and are located predominantly in the subepicardium of the ventricle [12, 13]. The central conduction system, which includes the sinoatrial node, atrioventricular node, and His bundle, is extensively innervated compared to the working myocardium [13-16]. We, and others, report that regional differences in cardiac sympathetic innervation, known as innervation patterning, are highly conserved among species [13, 14, 17].

The cardiac nervous system also involves afferent nerves. The sensory signals generated in the heart are conducted through cardiac afferent nerves, primarily thinly myelinated Aδ-fibers and nonmyelinated C-fibers [18, 19]. The sensory nerve fibers project to the upper thoracic dorsal horn *via* dorsal root ganglia neurons [18, 19]. Unlike sympathetic innervation, the anatomy of cardiac sensory innervation was poorly characterized until our recent report [20]. Cardiac sensory innervation will be discussed in more detail later in this review. 

## NERVE GROWTH FACTOR UPREGULATION CAUSES NERVE SPROUTING AND SUDDEN CARDIAC DEATH

Nerve growth factor (NGF) is a prototypic member of the neurotrophin family, the members of which are critical for the differentiation, survival, and synaptic activity of the peripheral sympathetic and sensory nervous systems [21-23]. The level of NGF expression within innervated tissues corresponds to innervation density [24].

Our recent investigations, and those of others, show that NGF expression is altered in diseased hearts [1-3, 25]. Studies in animal models by Zhou *et al*. [26] reveal that NGF is upregulated following myocardial infarction (MI), resulting in the regeneration of cardiac sympathetic nerves and heterogeneous innervation. In a previous study we report that NGF is upregulated in cardiac hypertrophy, leading to sympathetic hyperinnervation and rejuvenation [27]. NGF infusion after MI enhances myocardial nerve sprouting and results in a dramatic increase in SCD and a high incidence of ventricular tachyarrhythmia [1]. These results demonstrate that NGF-induced augmentation of sympathetic nerve sprouting in diseased hearts leads to lethal arrhythmia and SCD. 

## NERVE GROWTH FACTOR DOWNREGULATION IS CRITICAL FOR DIABETIC NEUROPATHY AND SILENT MYOCARDIAL ISCHEMIA

In contrast to sympathetic innervation, very little was known about sensory innervation and its alteration in diseased hearts. Visceral organs, including the heart, are believed to be rich in autonomic efferent innervation and poor in nociceptive afferent nerves [28]. Zahner *et al*. [29] report that vanilloid receptor-1-immunopositive sensory nerves are enriched in the epicardium and scarce in the myocardium. In immunohistochemical studies using anti-calcitonin gene-related peptide antibody (CGRP), a sensory marker, we demonstrated, for the first time, that cardiac sensory innervation is rich at epicardial sites and in the ventricular myocardium [20, 30]. In a screen of several neurotrophic factors, we found that the development of cardiac sensory nerves parallels the production of NGF in the heart [31]. Cardiac nociceptive sensory nerves that are immunopositive for CGRP, including the dorsal root ganglia and the dorsal horn, are markedly retarded in NGF-deficient mice and rescued in mice overexpressing NGF specifically in the heart. Thus, NGF synthesis in the heart is critical for the development of the cardiac sensory nervous system [32].

**Fig. (1). NGF gene transfer restores impaired sensory innervation in diabetic hearts.**
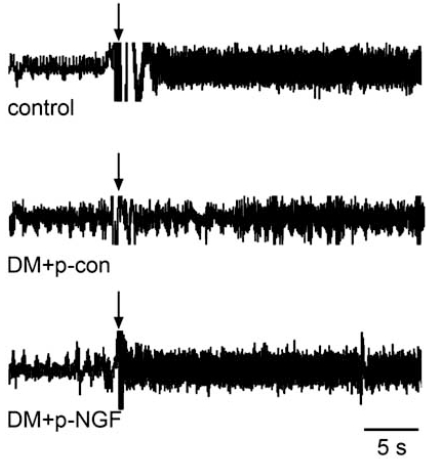
Recording of impulse activity from cardiac sympathetic afferent nerves. Myocardial ischemia was induced at the time point indicated by arrows. Note that the response of cardiac afferent nerves was reduced in DM injected with p-con (control). The gene transfer of 50 μg p-NGF preserved cardiac sensory nerve function in DM.

The cardiac sensory nervous system is responsible for pain perception and in the initiation of the protective cardiovascular response during myocardial ischemia [18, 19, 33, 34]. Cardiac sensory nerve impairment causes silent myocardial ischemia, a major cause of sudden death in DM patients [10]. Despite the severity of this complication, the alterations in cardiac sensory innervation and the molecular mechanism underlying sensory neuropathy in diabetic hearts is unclear [35-41]. To investigate whether NGF is involved in diabetic neuropathy, DM was induced with streptozotocin in wild-type (WT) mice and in transgenic mice that overexpressed NGF in the heart [20, 42-45]. Downregulation of NGF, CGRP-immunopositive cardiac sensory denervation, and atrophic changes in dorsal root ganglia were observed in DM-induced WT mice, whereas these deteriorations were rescued in DM-induced NGF-transgenic mice. Cardiac sensory function, measured by myocardial ischemia-induced c-Fos expression in dorsal root ganglia, was also downregulated by DM in the WT mice, but not in the NGF-transgenic mice [19]. Direct gene transfer of NGF into the diabetic rat hearts improved cardiac sensory inner-vation and function according to the electrophysiological activity of cardiac afferent nerves during myocardial ische-mia (Fig. **1**) [46, 47]. These findings demonstrate that DM-induced downregulation of NGF may lead to cardiac sensory neuropathy.

Phase I and phase II clinical trials of systemic administration of recombinant NGF reveal the safety and potential efficacy of this treatment for diabetic polyneuropathy, although a phase III trial showed no beneficial effects, possibly because the dosage and route of administration was suboptimal [48, 49]. It is possible that restriction of NGF dosage due to side-effects and the development of anti-NGF antibodies contributed to the lack of beneficial effects in the phase III clinical trial. To avoid these complications, gene transfer was used to directly administer the *NGF* gene into the heart. Both NGF- and CGRP-immunopositive nerves were reduced in diabetic hearts, thereby demonstrating the successful treatment of cardiac sensory neuropathy by *NGF* gene transfer. Consistent with our findings, the efficacy of *NGF *gene therapy has been reported in diabetic cystopathy and neuropathy of the footpad [49, 50]. Future studies on the reliability and efficacy of *NGF *gene therapy are required before clinical trials can proceed.

## ENDOTHELIN-1-INDUCED NGF UPREGULATION IS ESSENTIAL FOR CARDIAC SYMPATHETIC INNERVATION

Although alteration in the level of NGF has a great impact on the clinical outcome in heart disease, the molecular mechanisms underlying the regulation of NGF expression and sympathetic innervation are poorly understood. To address this issue, we performed a screen of several cardiac hypertrophic factors and found that ET-1 specifically upregulates NGF expression in primary cultured cardiomyocytes [51]. Furthermore, we showed that ET-1-induced NGF augmentation was not observed in cardiac fibroblasts but was specific to cardiomyocytes, and identified signaling molecules involved in the ET-1/NGF pathway.

**Fig. (2). Cardiac-specific overexpression of NGF overcomes the defects of cardiac sympathetic nervous system in Edn1-/- mice.**
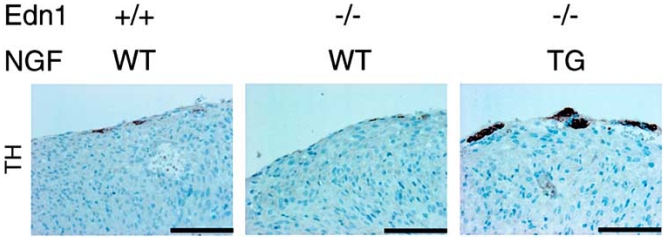
Immunostaining for TH in the hearts of Edn1^+/+^, Edn1^-/-^ and Edn1^-/-^/MHC-NGF mice. The reduced sympathetic innervation in Edn1^-/-^ hearts is rescued in Edn1^-/-^/MHC-NGF hearts. Scale bar, 100 µm.

To study the effects of the ET-1/NGF pathway on the development of the cardiac sympathetic nervous system, we analyzed various gene-modified mouse models. NGF expression, cardiac sympathetic innervation, and norepinephrine concentration were reduced in ET-1-deficient mouse (*Edn1*^-/-^) hearts, but not in the hearts of angiotensinogen-deficient mice (*Atg*^-/-^). In *Edn*1^-/-^ mice, the sympathetic stellate ganglia exhibited excessive apoptosis and displayed loss of neurons at the late embryonic stage [52]. Moreover, we demonstrated that cardiac-specific overexpression of NGF in *Edn*1^-/-^ mice overcomes sympathetic nerve retardation (Fig. **2**) [53]. These findings indicate that ET-1 is a key regulator of NGF expression in cardiomyocytes, and that the ET-1/NGF pathway is critical for sympathetic innervation in the heart [54]. Given that ET-1 is strongly induced in myocardial infarction and cardiac hypertrophy, the ET-1/NGF pathway may also be involved in NGF upregulation and nerve regeneration in pathological hearts.

## CARDIAC SYMPATHETIC INNERVATION PATTERNING IS DETERMINED BY SEMA3A EXPRESSION

The growth-cone behavior of nerves is determined by coincident signaling between neural chemoattractants and chemorepellents, synthesized in the innervated tissue. While NGF plays critical roles in cardiac innervation as a chemoattractant, the neural chemorepellent that induces growth-cone collapse and repels nerve axons is not found in the heart. Recently, we found that Sema3a inhibits neural growth and establishes appropriate innervation patterning in the heart [55].

**Fig. (3). Inverse expression pattern of Sema3a and sympathetic innervation in mouse hearts.**
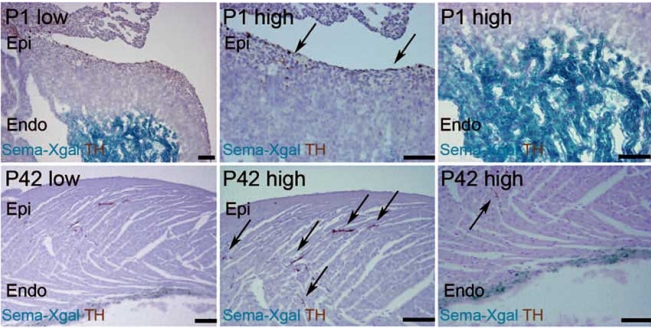
Double staining with TH (brown) and X-gal (blue) in P1 and P42 Sema3a^*lacZ*/+^ hearts. Sympathetic nerves are restricted to the subepicardium at P1, but extend into the myocardium, coincident with downregulation of Sema3a at P42. Scale bar, 100 µm.

Sema3a is a Class 3 secreted semaphorin and has been cloned and identified as a potent neural chemorepellent and a directional guidance molecule for nerve fibers [56-58]. For this reason, investigations were initiated to determine if cardiomyocytes produced Sema3a and if this protein played a role in sympathetic neural patterning and cardiac performance. We analyzed the kinetics and distribution of cardiac sympathetic innervation in developing mouse ventricles [55]. Sympathetic nerve endings, immunopositive for the sympathetic marker, tyrosine hydroxylase (TH), appeared on the epicardial surface at embryonic day (E)15 and then gradually increased in number in the myocardium after postnatal day (P)7 and P42. Sympathetic nerves were more abundant in the subepicardium than in the subendocardium of the ventricular myocardium, suggesting the presence of an epicardial-to-endocardial gradient that is consistent with previous reports [12, 14-16]. We analyzed heterozygous *Sema3a* knocked-in *lacZ* mice (*Sema3a^lacZ/+^*) to investigate the expression pattern of *Sema3a* and its relationship to innervation patterning in the heart. At E12, strong *lacZ* expression was detected in the heart, especially in the trabecular components of the ventricles. By E15, *lacZ* expression was observed in the subendocardium, but not in the subepicardium, of the atria and ventricles. At P1 and P42, *lacZ* expression was reduced in certain regions and highlighted the Purkinje fiber network along the ventricular free wall [59, 60]. Quantitative RT-PCR of *Sema3a* in developing hearts revealed a linear decrease in the expression of *Sema3a* from E12 that corresponded with an increase in sympathetic innervation density. The negative correlation between the kinetics of *Sema3a* expression and sympathetic innervation indicates that Sema3a negatively regulates cardiac innervation in developing hearts (Fig. **3**).

## SEMA3A DEFICIENT MICE SHOW SINUS ARREST AND SUDDEN DEATH

To investigate whether Sema3a is critical for cardiac sympathetic nerve development, we analyzed *Sema3a-*deficient mice (*Sema3a^–/–^*) [55, 61, 62]. The WT hearts showed a clear epicardial-to-endocardial gradient of sympathetic innervation. By comparison, the sympathetic nerve density was reduced in the subepicardium and enhanced in the subendocardium of *Sema3a^–/– ^*mice, resulting in disruption of the innervation gradient in *Sema3a^–/–^* ventricles. The *Sema3a^–/–^* mice also exhibited malformation of the stellate ganglia that extend sympathetic nerves to the heart.

**Fig. (4). Various arrhythmias occurred in Sema3a gene-modified mice.**
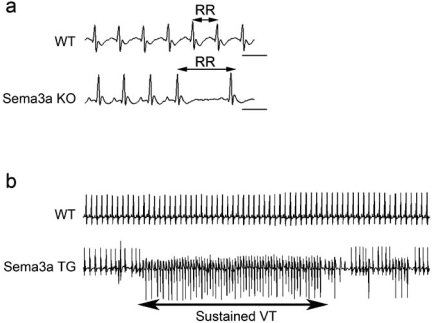
(a) ECG in WT and *Sema3a*^–/–^ mice. Note the abrupt sinus-slowing in *Sema3a*^–/–^ mice. (b) Epinephrine administration revealed sustained VT only in *SemaTG* mice.

Strikingly, we also found that most of the* Sema3a^–/–^* mice died within the first postnatal week, with only 20% surviving until weaning [55, 61, 62]. To identify the cause of death and the effects of abnormal sympathetic neural distribution in* Sema3a^–/–^* hearts, we performed telemetric electrocardiography and heart-rate variability analysis [63, 64]. In addition to multiple premature ventricular contractions, *Sema3a^–/–^* mice developed sinus bradycardia and abrupt sinus arrest due to sympathetic neural dysfunction (Fig. **4**).

## SEMA3A-OVEREXPRESSION CAUSES VENTRICULAR TACHYARRHYTHMIAS DUE TO INNERVATION PATTERNING DEFECTS

We generated transgenic mice that overexpressed *Sema3a* specifically in the heart (*SemaTG*) to determine if the phenotype observed in *Sema3a^–/– ^*hearts is a secondary effect of stellate ganglia malformation [65]. This possibility was discounted as *SemaTG* mice showed reduced sympathetic innervation and attenuation of the epicardial-to-endocardial innervation gradient.

**Fig. (5). Cardiac innervation patterning and lethal arrhythmias.**
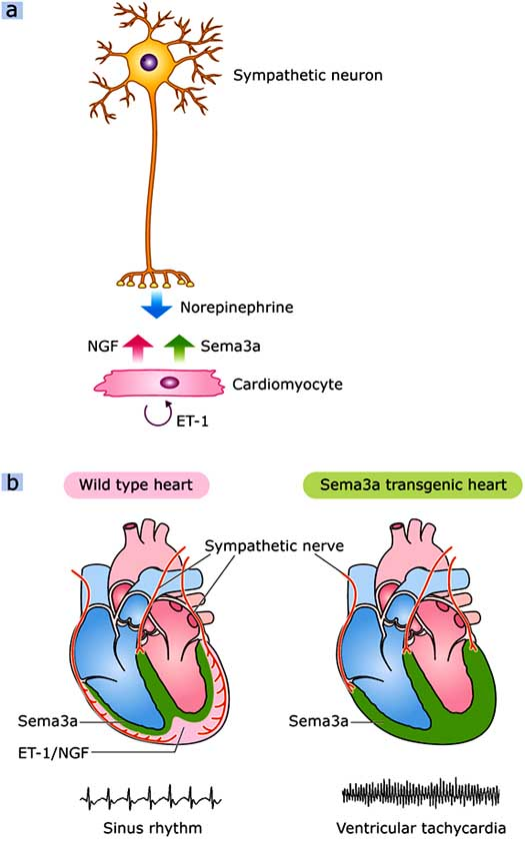
The crosstalk between cardiomyocytes and sympathetic neurons. Neurons synthesize norepinephrine, and cardiomyocytes secret NGF and Sema3a to control cardiac performance. (b) Appropriate Sema3a-mediated sympathetic innervation patterning is critical for the maintenance of an arrhythmia-free heart. *SemaTG* mice are highly susceptible to ventricular tachyarrhythmias.

The *SemaTG* mice died suddenly without symptoms at 10 months of age. Sustained ventricular tachyarrhythmia was induced in *SemaTG* mice, but not in WT mice, after epinephrine administration, and programmed electrical stimulation revealed that *SemaTG* mice were highly susceptible to ventricular tachyarrhythmia (Fig. **4** and **5**) [66, 67]. The β1-adrenergic receptor density was upregulated and the cAMP response after catecholamine injection was exaggerated in *SemaTG* ventricles. Action potential duration was significantly prolonged in hypoinnervated *SemaTG* ventricles, presumably via ion channel modulation. These results suggest that the higher susceptibility of *SemaTG* mice to ventricular arrhythmia is due to catecholamine super-sensitivity and prolongation of action potential duration, both of which can augment triggered activity in cardiomyocytes [68-72]. Thus, Sema3a-mediated sympathetic innervation patterning is critical for the maintenance of arrhythmia-free hearts.

## CONCLUSIONS

Cardiac nerves are highly plastic, and the balance between NGF and Sema3a synthesized in the heart determines cardiac innervation patterning (Fig. **5**). ET-1 upregulates NGF expression in cardiomyocytes, modulates nerve sprouting and plays critical roles in sympathetic nerve development [27, 54]. NGF is also important for sensory innervation, and NGF downregulation may result in silent myocardial ischemia and SCD in diabetic patients [20]. On the other hand, Sema3a inhibits neural growth and establishes appropriate innervation patterning in the heart. Both Sema3a deficiency and overexpression reveal lethal arrhythmias and sudden death due to disruption of sympathetic innervation patterning, suggesting fine tuning of Sema3a is critical for cardiac function. Thus, identification of the molecular mechanisms regulating cardiac innervation would improve our general understanding of cardiac performance and disease.
